# Along urbanization sprawl, exotic plants distort native bee (Hymenoptera: Apoidea) assemblages in high elevation Andes ecosystem

**DOI:** 10.7717/peerj.5916

**Published:** 2018-11-07

**Authors:** Patricia Henríquez-Piskulich, Alejandro Vera, Gino Sandoval, Cristian Villagra

**Affiliations:** 1 Instituto de Entomología, Universidad Metropolitana de Ciencias de la Educación, Santiago, Región Metropolitana, Chile; 2 Departamento de Biología, Universidad Metropolitana de Ciencias de la Educación, Santiago, Región Metropolitana, Chile; 3 Departamento de Historia y Geografía, Universidad Metropolitana de Ciencias de la Educación, Santiago, Región Metropolitana, Chile

**Keywords:** Exotic plant species, Apoidea, Montane ecosystems, Pollinators, Native bee assemblage, Urbanization

## Abstract

Native bees contribute a considerable portion of pollination services for endemic as well as introduced plant species. Their decline has been attributed to several human-derived influences including global warming as well as the reduction, alteration, and loss of bees’ habitat. With human expansion comes along the introduction of exotic plant species with negative impacts over native ecosystems. Anthropic effects may even have a deeper impact on communities adapted to extreme environments, such as high elevation habitats, where abiotic stressors alone are a natural limitation to biodiversity. Among these effects, the introduction of exotic plants and urbanization may have a greater influence on native communities. In this work, we explored such problems, studying the relationship between the landscape and its effect over richness and abundance of native bees from the subandean belt in the Andes mountain chain. Furthermore, we investigated the effects of exotic plant abundance on this high-altitude bee assemblage. Despite the landscape not showing an effect over bee richness and abundance, exotic plants did have a significant influence over the native bee assemblage. The abundance of exotic plants was associated with a relative increase in the proportion of small and medium bee species. Moreover, Halictidae was the only family that appeared to be favored by an increase in the abundance of exotic plant species. We discuss these results and the urgent need for further research of high-altitude environments due to their vulnerability and high endemicity.

## Introduction

Native bee species are not only important as native plant pollinators, but also 15–30% of productive crops require their pollination services (McGregor, 1976). Declines of these insects have been reported at a global scale (Biesmeijer et al., 2006; Fitzpatrick et al., 2007; Cameron et al., 2011). Human activities are the main drivers of native bee’s downturn through: fragmentation, biotic homogenization with the introduction of invasive organisms (McKinney & Lockwood, 1999), as well as insect’s habitat quality degradation and, ultimately, destruction (Foley et al., 2005; Potts et al., 2010). Furthermore, climate change has also been listed among the explanations for native bee declines (Potts et al., 2010). Among the consequences of pollinator declines are changes in the structure of biotic communities, degradation of biodiversity, and reduction of food production from negative effects on native flowers and crops of economic importance that depend on pollinators to achieve reproduction (Allen-Wardell et al., 1998; Potts et al., 2010). Pollinator declines may not be easy to recover from if they continue progressing from the continuous anthropogenic pressure on pollinators (Allen-Wardell et al., 1998; Winfree et al., 2009).

This situation could be even more critical for native insects adapted to extreme environments, including bees. At a local scale, insects may be especially sensitive to human impact in habitats with extreme climate fluctuations (Boggs & Murphy, 1997; Haslett, 1997). This could be the case of high elevation environments, such as montane ecosystems, also recognized as hotspots of biological diversity (Lomolino, 2001). Under these conditions, in addition to overcoming current human impacts, native bees must face extreme environmental variation such as severe changes in temperature, elevated levels of ultraviolet radiation, less partial pressure of atmospheric gases, drastic oscillations in the amount of precipitations, strong wind speed, among others (Hodkinson, 2005). All these abiotic factors have been associated to comparatively reduced diversity and specialized insects in high-altitude habitats (Pellissier et al., 2012; Classen et al., 2015). In general, studies have found lower richness and abundance of insects as altitude increases (Hägvar, 1976; Wolda, 1987; Lefebvre et al., 2018), and, regarding native bee species, the same pattern has been reported (Arroyo, Primack & Armesto, 1982; Hoiss et al., 2012).

With economic development, it often increases environment degradation in favor of urbanization, especially in countries with unsustainable development policies (Romero & Ordenes, 2004). This problem may also impact high-altitude ecosystems (Baiping et al., 2004; Romero & Ordenes, 2004). Regarding the consequences of urbanization on native bee habitats, it has been demonstrated that this affects bee richness and evenness, and also, delays peak abundance during the bee season and decreases temporal turnover (Winfree, Bartomeus & Cariveau, 2011; Hung, Ascher & Holway, 2017). Also, the landscape modifications have been found to affect bee assemblages due to species-specific responses such as: body size, nesting habits, feeding behavior and sociality level, among others (Bishop & Armbruster, 1999; Williams et al., 2010; Hopfenmüller, Steffan-Dewenter & Holzschuh, 2014; Marshall et al., 2015). Considering this, there could be an array of different responses: Some species could respond positively while others could be under threat of disappearing from an ecosystem (Hinners, Kearns & Wessman, 2012; Fortel et al., 2014). Bees with feeding specializations (also called “oligolectic”) gather resources on floral species of one family or genera of plants, and therefore, are less flexible to changes in their habitats due to human-derived modifications (Steffan-Dewenter et al., 2006; Hernandez, Frankie & Thorp, 2009). For instance, changes in the landscape decrease the proportion of parasitic (Steffan-Dewenter et al., 2006), solitary and also larger-sized native bee species (Hinners, Kearns & Wessman, 2012). Contrastingly, urbanization may favor cavity-nesting species (Fortel et al., 2014) probably due to the higher nesting resources for these species in urbanized areas (Hernandez, Frankie & Thorp, 2009). Therefore, knowledge of the pollinator’s resources and life-history traits is required to correctly assess the most likely pollination responses under the effects of human activities (Cane et al., 2006).

The impact of exotic plants over native bee species has been poorly studied (Corbet et al., 2001; Goulson, 2003; Liu & Pemberton, 2008). To the best of our knowledge, the effects of exotic plant species on mountain native bee assemblages are yet to be revealed. Their influence could be particularly relevant if they are capable of modifying the landscapes and native plant communities, due to cascading effects on different trophic levels (Morón et al., 2009; Fenesi et al., 2015). In high elevation environments it has been suggested that exotic plants may jeopardize native pollination services (Miller et al., 2018) and potentially affect native bee populations through modifying the relative abundance and the diversity of native plant species (Stout & Morales, 2009). Previous studies have found a negative relationship between the presence of exotic plants and the abundance, species richness, and evenness of native bees (Morón et al., 2009; Fenesi et al., 2015). However, contrary to general trends in landscape studies, there are no works on the effect of exotic plants over the species-specific response of native bees due to the threats previously mentioned. If exotic plant species could alter mountain bee assemblages, this may have significant effects on pollination of native plant species. Since their effect could vary depending on which bee species is considered, some native bee species could benefit from the introduction of exotic plants, as providers of additional resources (Tepedino, Bradley & Griswold, 2008; Drossart, Michez & Vanderplanck, 2017). Nonetheless, it has been recently demonstrated that the introduction of exotic plants could be even more problematic that changes in the landscape, affecting not only insect assemblages but also complete plant-pollinator networks (Hansen et al., 2017). If exotic plants replace the majority of native plant species, not only bees would face the consequences of this introduction, but also the whole ecosystem services may be hampered due to alterations to the biota (Wilde, Gandhi & Colson, 2015).

Despite above-mentioned problems, we found no published studies that focus on the response of native bee assemblages towards the effects of the landscape’s changes and the introduction of exotic flora in high elevation ecosystems. These dimensions could be essential for the understanding of bee declines, and preventing further losses not only of these insects, but also the rest of pollinating animals (Aguirre‐Gutiérrez et al., 2015). In this work, we evaluated if urbanization could have an effect on native bee species richness and abundance. In addition, we focused on the influence of the abundance of exotic plant species and the response of native bee assemblages in order to evaluate species-specific responses. In general, there is still much to explore from montane biomes (Lomolino, 2001). In this context, previous works have listed nearly 50 species of native bees for the subandean belt of central Chile, an area that unfortunately is under constant alteration due to the replacement of natural habitats by urban expansion (Arroyo, Primack & Armesto, 1982; Camousseight & Barrera, 1998; Monckton, 2016). In particular, we tested the hypothesis that urbanization mediates changes in diversity, and also, that the introduction of exotic flora that comes along with this, could also play a role in changing the assemblage due to the close relationship between bees and their floral resources. Our objectives were to assess the effect of urbanization over high-altitude native bee diversity and of exotic floral abundance over the assemblage.

## Materials and Methods

### Study site

We carried out our study in the town of Farellones and its surroundings (33°20′59″S, 70°18′34″O), located at 2,360 m above sea level in the subandean vegetational belt of Andes mountains of central Chile. A zone characterized by long, snow free-periods of 5–8 months, and annual precipitations of 400–800 mm falling mostly as snow during winter (Arroyo, Armesto & Villagran, 1981). Corresponds to a settlement started around 1930s as a winter sport center and presently the larger ski destination in Chile, with an ongoing expansion of urban areas (Junta de Vecinos de Farellones, 2018). Moreover, as in other urbanized high elevation sites along Andes mountain chain, it is possible to find livestock seasonal foraging activities as well as mining exploitation routes (Bahre, 1979; SERPLAC (Secretaría Regional de Planificación y Coordinación), 1980).

In this locality, vegetation is represented by subandean scrub, mainly composed by the Asteraceae family, accompanied by perennial herbs, geophytes, and annual herbs (Arroyo, Primack & Armesto, 1982). It has been described that 54% of its vascular flora is native and 29% endemic (Muñoz-Schick et al., 2000).

We selected eight sites for this study of 80 × 80 m (Fig. S1), criteria for this selection were: (i) the vegetation was unmanaged; (ii) sites were exposed to human activities; and (iii) safe enough to sample, considering that the area presented cliffs and sharp edge precipices. Data was collected from the sites once a month for two seasons: the first in December 2016, January and February 2017 (season 1: 2016/2017), and the second in November and December 2017, January and February 2018 (season 2: 2017/2018). Weather with abundant snow precipitation did not permit us to sample in November 2016. Minimal and maximal distances between sites were 380 m and 4.4 Km, respectively (Normandin et al., 2017).

### Plants

In order to determine native and exotic floral abundance for each month sampled, we defined four transects of 80 m long × 2 m wide, covering approximately 10% of the total site area. In these transects all flowers of blooming species encountered within one meter on each side was counted. Later, we calculated total density of native and exotic plant for each month. For each season, we obtained an average of native and exotic floral abundance per month sampled for every site. In addition, for each site we calculated the percentage covered by urban landscape through the analysis of aerial photographs taken with a drone, with ArcGIS v 10.5 to avoid bias caused by the possible effect of other factors associated with human activities. Proximity of each site to urban settlements and roads measured in meters was also considered a factor of human impact. Furthermore, since our study was done in a high mountain environment, altitude of each site was recorded.

### Bee sampling

We sampled bees on sunny days, with temperatures over 15 °C and winds below 15 Km/h (following Fortel et al., 2014). Pan traps and insect nets were used to assess bee assemblages (Nielsen et al., 2011). Pan traps corresponded to plastic bowls painted with yellow, blue, or white fluorescent paint (Rocol Top, Asnières-sur-Seine, France) (Normandin et al., 2017). For these samplings, we defined three triplets of pan traps per site. In each triplet we considered one of every color used. These recipients were filled with 400 mL of water and a drop of detergent. Pan traps were separated from each other by 20–40 m forming a three m side equilateral triangle that we left to work at each site from 9:00 to 17:00 (Westphal et al., 2008).

Complementarily, active net sampling took place one hour during the morning (9:00–12:00) and one hour during the afternoon (15:00–17:00), in order to cover for the majority of bee activity for this mountain habitat (LeBuhn et al., 2003). Specimens collected were first stored in 70% ethanol, and later washed, processed, pinned, and identified to the lowest taxonomic level using several keys and specialized taxonomist assistance (Chiappa, Rojas & Toro, 1990; Toro & Rodríguez, 1998; Toro, 1997, 1999; Monckton, 2016). For specimens identified only to genus level, we made sure they corresponded to the same species. For *Bombus dahlbomii* Guérin-Méneville, 1835 (Hymenoptera: Apidae), an endangered (Morales et al., 2016) and conspicuous species, we only collected them to take into account the relative abundance in each sampled site and after the 1-h sampling period they were all released. Introduced species, such as *Apis mellifera* and *Bombus terrestris*, were not collected in this study.

### Data analysis

We computed the parameters separately for the two sampled seasons because of the difference in sampling effort and climatic variability (e.g., precipitation and maximum temperatures) (Vuille, 2014). During the first season we were unable to sample in November due to bad weather conditions. Moreover, Central Chile and Argentina near Andes zones are highly affected by “el niño” and “la niña” climatic oscillations (Cortes & Margulis, 2017; Malmros et al., 2018). Species diversity was characterized by species richness in EstimateS (version 9.1.0; Colwell, CT, USA). We computed the observed cumulative species richness curve and the total expected species richness with a bootstrapping of 1,000 random iterations of sampling order. In regards to total expected species richness, we used Chao2 since it is the least biased estimator (Gwinn et al., 2016).

Regarding native bee diversity, to assess correlation between the landscape variables we used Spearman correlation coefficients in SPSS, in order to avoid the effect of outliers and biased correlation results (Suchowski, 2001). Later, for each sampled season, we evaluated the effect of the landscape variables on native bee richness and abundance through generalized linear models (GLM), using glm function in R. Since the dependent variables of richness and abundance presented overdispersion, we used a negative binomial model to take this into account. Furthermore, the effect of each landscape variable was nested in the season to account for inter-seasonal variation. Considering the results of correlation analyses, we maintained the model with the variable of the correlation set with the lowest Akaike Information Criterion (AIC) value, regarding it as the most parsimonious alternative (Johnson & Omland, 2004).

For the native bee assemblage of our study, we wanted to relate the functional traits of native bees with the abundance of exotic flora. First, we classified our collected native bee specimens based on different functional traits at species level into: “parasitic” and “non-parasitic.” Afterward, the “non-parasitic” group was subdivided by feeding behavior into: “oligolectic” or “polylectic.” This was done because adults of parasitic bees forage only for nectar (Roubik, 1989; Heard, 2016). This classification was based on previous published information (Jaffuel & Pirión, 1926; Ruiz, 1944; Rozen, 1967; Wagenknecht, 1969, 1970; Ehrenfeld & Rozen, 1977). If there was no information available of functional traits of a particular bee species, we used the information available from the nearest related species.

We used the inter-tegular distance (ITD) as a proxy for body size and the functional trait of foraging distance on every individual collected (Greenleaf et al., 2007). Measures were done with the software tpsDig v 2.32, using photographs of the thorax of every collected specimen (Hoiss et al., 2012; Fortel et al., 2014). For *B. dahlbomii*, we measured the ITD from several specimens from Instituto de Entomología, UMCE collection. Species were then grouped into small (<1.5 mm), medium (1.5–3 mm), and large (>3 mm) size classes (Hinners, Kearns & Wessman, 2012).

To determine how groups of species that shared above-mentioned functional traits responded to exotic floral abundance, in each sampled site we tested for the proportion of each functional trait in regards to the site’s exotic floral abundance. We pooled the data from both seasons and used the proportion of the total number of native bee individuals (abundance) and total number of species for each classification group. In addition, we evaluated the possible effect of exotic floral abundance in montane bee’s assemblage at the family level. For both, bee family and functional group analyses, to evaluate the response of exotic floral abundance we used multinomial and binomial logistic regression models with JMP (version 14; SAS Institute, Cary, NC, USA) (Hinners, Kearns & Wessman, 2012).

We applied the threefold Bonferroni correction (Rice, 1989) along the three functional trait categories for these analyses to control for a high probability of obtaining significant results due to chance because of the number of non-independent tests we conducted (Rice, 1989).

## Results

### Native bee diversity

**Figure 1:**
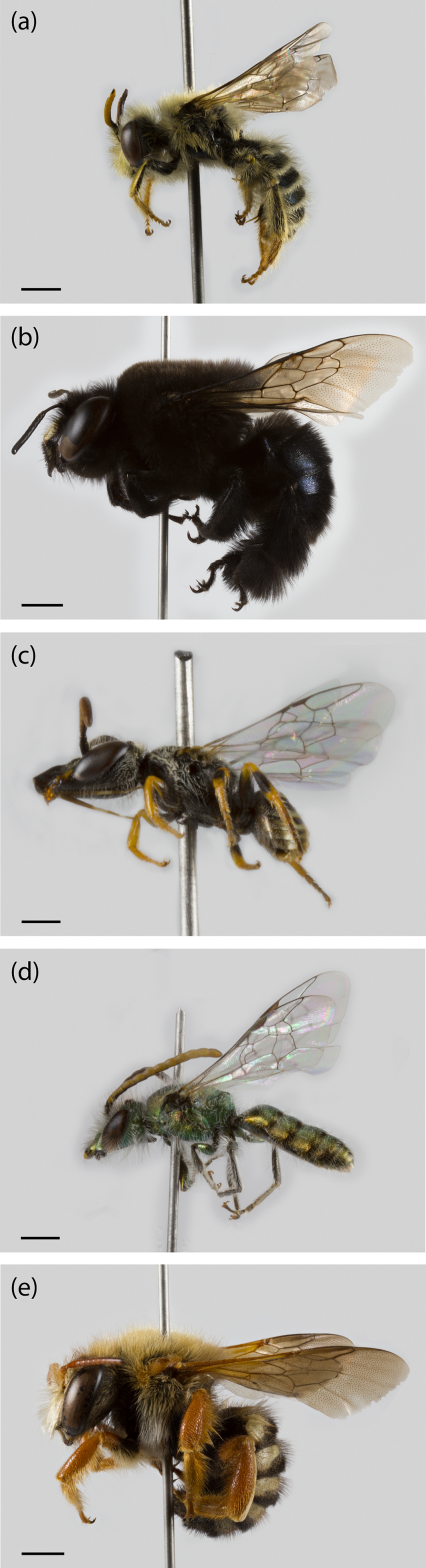
Specimens from the different native bee families collected during the study in Farellones, Chile. (A) *Acamptopoeum submetallicum* (Andrenidae), scale bar 1.5 mm. (B) *Centris nigerrima* (Apidae), scale bar two mm. (C) *Xeromelissa* sp. (Colletidae), scale bar 0.5 mm. (D) *Caenohalictus iodurus* (Halictidae), scale bar one mm. (E) *Anthidium chubuti* (Megachilidae), scale bar two mm. Photography: Patricia Henríquez-Piskulich.

**Figure 2:**
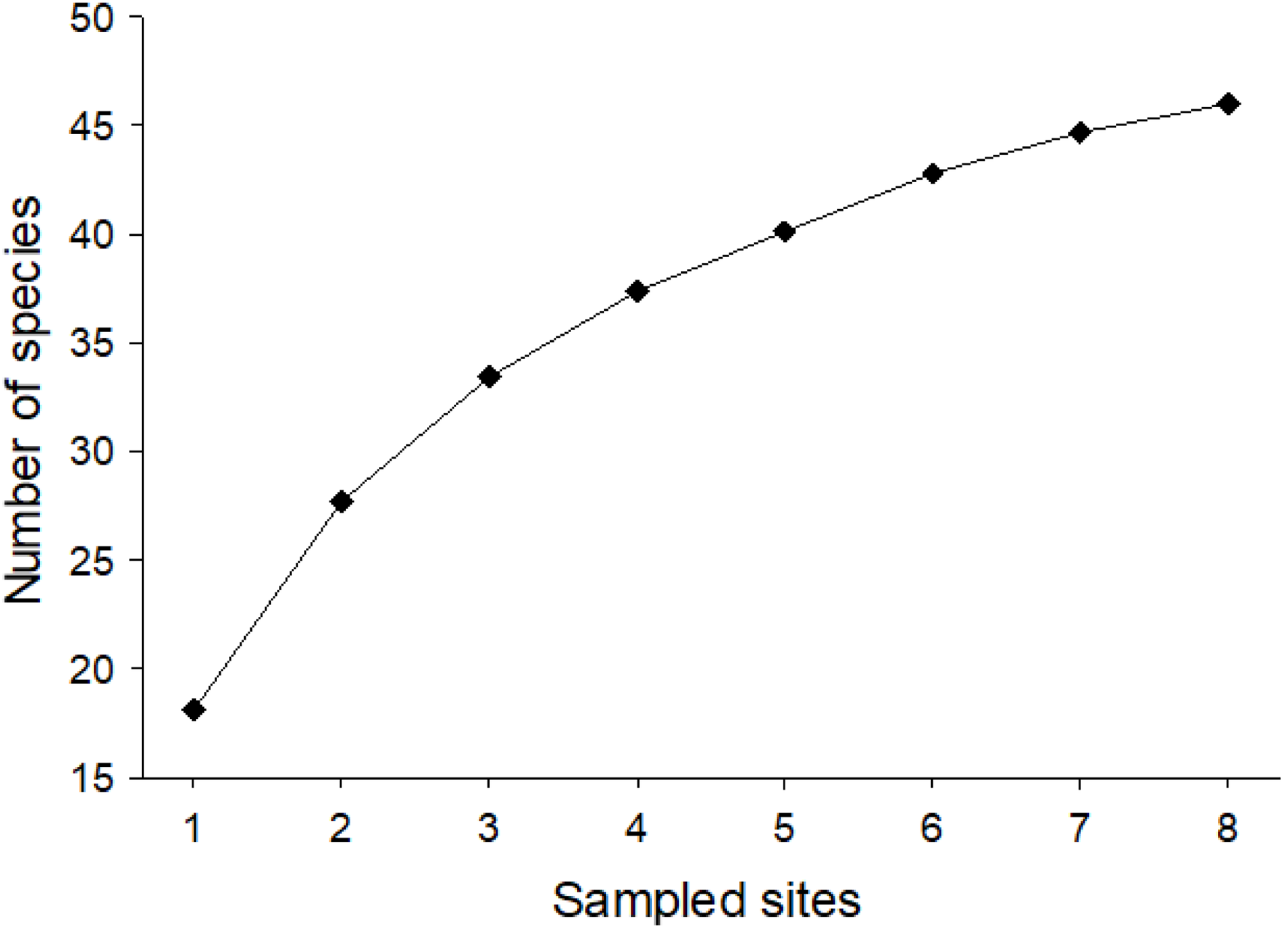
Mean species accumulation curve for pooled data of native bees collected for the two sampled seasons of the study (1,000 randomizations). The vertical axis corresponds to number of species and the horizontal to sampled sites.

Considering the two sampling seasons of 2016/2017 and 2017/2018, a total of 1,052 bee specimens were collected, 212 in season 1 and 840 in season 2. In total, this corresponded to 28 genera and 46 species (32 in 1 and 40 in 2) with a minimum of nine species and a maximum of 27 species per site (Table S1). They belonged to five families: Andrenidae (seven species), Apidae (13 species), Colletidae (seven species), Halictidae (11 species), and Megachilidae (eight species) (Fig. 1). Nonetheless, after our 2-year study, it was still not possible to ensure that we had collected all the potential species from Farellones, which can be confirmed by the species accumulation curve not reaching saturation (Fig. 2). Estimated species richness of both pooled seasons was 52.83, therefore, approximately 87.07% of native bee species present in our study location were collected during our work (Table 1).

**Table 1:** Observed and estimated species richness for each sampled season and pooled data.

Season	Sobs^a^ ± Sd^b^	Chao 2 ± SD (completeness)
2016–2018	46 ± 9.53	52.83 ± 5.15 (87.07)
2016/2017	32 ± 8.07	43.48 ± 7.98 (73.60)
2017/2018	40 ± 8.29	46.62 ± 5.29 (85.80)

**Notes:**

The first column corresponds to the analyses of both pooled seasons and each season evaluated separately. The second, to observed species richness and its standard deviation. Finally, the third column represents Chao2 estimator, its standard deviation and the completeness of native bee sampling.

a Sobs, observed species richness.

b Sd, standard deviation.

For both seasons 12 bee species (26.09%) were recorded as singletons and four (8.70%) as doubletons. In regards to singletons, two species (4.35%) were parasitic. In total, five species (10.87%) were parasitic and 41 non-parasitic. Non-parasitic were mostly polylectic (78.05%). From the 46 species collected, 15 represented from 1.14% to 5.80% of the total number of specimens (12–61 specimens). The three most abundant species were: *Lasioglossum* sp. (279 specimens; 26.52% of the total), *B. dahlbomii* (121 specimens; 11.50%), and *Caenohalictus iodurus* (117 specimens; 11.12%), all of them are polylectic.

Spearman correlation coefficients showed for both seasons a negative correlation between distance to the nearest town and exotic floral abundance (*r* ≤ −0.857, *n* = 8, *p* ≥ 0.002) (Table S2). For the GLM of dependent variables, native bee richness and abundance, we only maintained distance to nearest town since the models with this variable gave the lowest AIC to explain both. Regarding these analyses, the landscape variables showed no significant effect over native bee richness and abundance (Table S3).

### Native bee species assemblage

Within the plant species found during our field work and used to evaluate changes in the bee assemblage, we encountered 39 plant species: 24.32% were exotic, 70.27% native, and 5.41% were endemic for Chile (Table S4).

**Figure 3:**
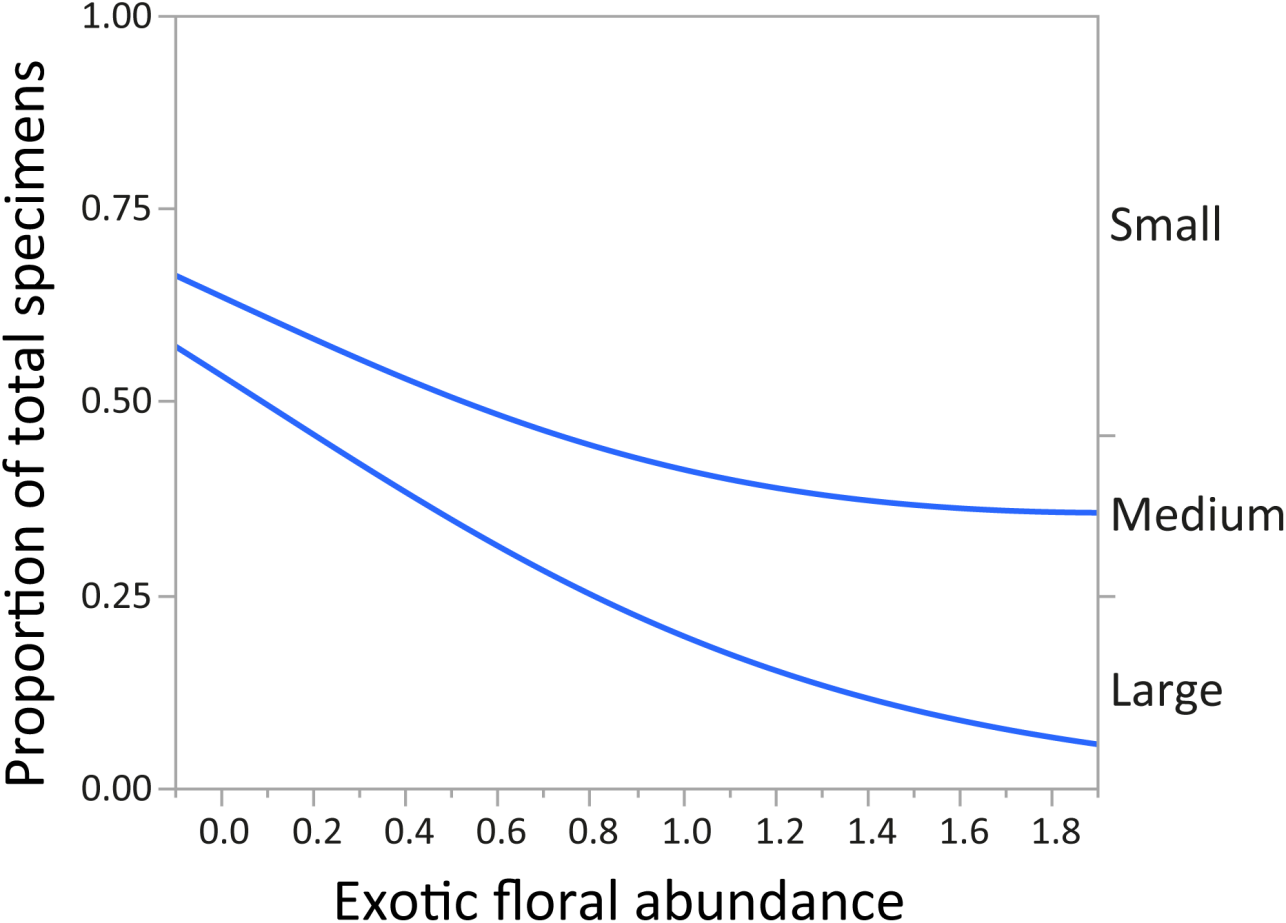
Changes in relative proportions of native bee species grouped by body size (small, medium, and large) with exotic floral abundance (χ^2^ = 197.96, *p* < 0.0001). Lines are least-squares regression lines depicting the proportions of each group. The left vertical axis corresponds to the proportion of total specimens and the horizontal to exotic floral abundance proportion. The groups of the right vertical axis correspond to areas between the regression lines.

In regards to mountain bee assemblage composition, “parasitism” and “feeding behavior” had no significant relationship with abundance of exotic plant species. After Bonferroni correction, the variable “body size” (by abundance) showed a significant relationship with “exotic floral” abundance. As the abundance of the latter increased, the proportion of small and medium native bee species was greater, and the proportion of large individuals decreased (*χ*^2^ = 197.96, *p* < 0.0001) (Fig. 3).

**Figure 4:**
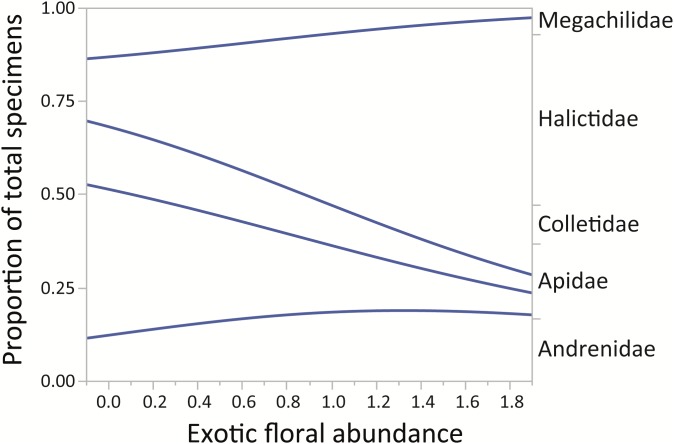
Changes in relative proportions of native bee species grouped by family (Andrenidae, Apidae, Colletidae, Halictidae, and Megachilidae) with exotic floral abundance (χ^2^ = 229.88, *p* < 0.0001). Lines are least-squares regression lines depicting the proportions of each group. The left vertical axis corresponds to the proportion of total specimens and the horizontal to exotic floral abundance proportion. The groups of the right vertical axis correspond to areas between the regression lines.

Finally, for the native bee families in the assemblage, the proportion of Halictidae increased while the proportions of Apidae, Colletidae, and Megachilidae decreased (*χ*^2^ = 229.88, *p* < 0.0001). The family Andrenidae maintained a relatively small proportion in all sampled sites (Fig. 4).

## Discussion

After our 2-year study in the subandean belt of montane Andes, we found that the introduction of exotic plant species did show an association with changes in native bee species assemblages. In sites with higher abundance of exotic plants the composition of native bees changed in regards to their body size (Fig. 3), and also at a taxonomic level (Fig. 4). Furthermore, we found no evidence of a possible effect of the landscape variables considered, represented by: distance to nearest town, distance to roads, percentage of urban landscape cover, altitude and native floral abundance, on to native bee richness and abundance (Table S3).

Even though our work included two seasons, we weren’t able to collect all the potential species present in our study location, as showed by the mean species accumulation curve. We found 46 species, which represents 87.07% of the predicted native bee species richness of this montane area (Table S1). We sampled each of the eight sites from this high Andean zone for a total of seven times (each survey done every 4 weeks during the bee season of both years) and obtained 12 species (26.09%) of bees in the form of singletons. Our number of singletons is in accordance with literature, where the average of singletons for bee studies is 28% (Williams, Minckley & Silveira, 2001).

Exotic floral abundance proved to be correlated with the proximity to urban areas, where a higher abundance of introduced plants individuals was found near urbanized lands. High elevation ecosystem may be very sensitive to human-derived changes such as the introduction of exotic species (Badano et al., 2007). It is possible to suggest that the problem lies in the biotic homogenization that comes along with urbanization, and the consequent replacement of native and endemic species by invasive and exotic ones (McKinney & Lockwood, 1999). Urbanization also reduces native flora and fauna diversity, and at the same time, promotes the reproduction, and colonization by exotic plant species (Marzluff, 2001; McKinney, 2002; Frankie et al., 2005). The latter has proven to have significant effects over many ecological variables, the problem falling in the varying magnitude and direction of these context dependent effects (Vilà et al., 2011). Nonetheless, exotic flora could in some cases decrease richness and abundance of native plant and insect species, and at the same time reduce insect biomass with lower insect productivity as a consequence (Heleno et al., 2009; Cook-Patton & Agrawal, 2014; Van Hengstum et al., 2014). For native bees, varying responses to exotic plant species have been found: where some species may be favored due to the alteration of plant community composition and structure, others may be unable to forage and complete their life cycles, due to their inability to use exotic plants (Stout & Morales, 2009). Therefore, some bee species may be especially sensitive to the loss of their native plants (McKinney, 1997), a problem that could be potentially important at high altitudes, where Hymenoptera are the dominant flower visitors (Arroyo, Primack & Armesto, 1982; Makrodimos et al., 2008). Studies have found a relationship between floral specialization and risk of extinction, were oligolectic species are at a higher risk of being affected by changes in their habitats (Packer et al., 2005; Roberts et al., 2011). Even though most bee species in our study were polylectic, loss of dominant plant species in an ecosystem might adversely affect generalists and specialists in the same manner (Frankie et al., 1997). Furthermore, since native plant richness of an ecosystem is negatively correlated with the vulnerability to plant invasions (Knops et al., 1999), mountain environments could be more susceptible to the dispersal of exotic plant species due to the decrease in species richness with altitude (Rahbek, 1995). This becomes very relevant not only because of the great endemism of its community (Muñoz-Schick et al., 2000), but also because there is already evidence supporting the classification as endangered for bee species in this habitat, such as the case of *B. dahlbomii*, the largest Apiformes known to date (Morales et al., 2016). A species that could already be threatened by the presence of the introduced bumblebee *B. terrestris* (Montalva, Ruz & Arroyo, 2008; Montalva, 2012; Arbetman et al., 2013).

For montane Andes, we found an association between exotic floral abundance increase and the rise in the proportion of small and medium native bee specimens (Fig. 3). On the other hand, the proportion of bee specimens of large sizes dropped along the increase of introduced plant species (Fig. 3). It is important to emphasize that in proportional data, when one or more group increases, other groups must decrease. Regarding the consequences of exotic flora over the phylogenetic structure of the assemblage, the effect may be probably related to body size, since Halictidae specimens present in our study ranged between small or medium sized bees and were the only family that showed a rise in its proportion of total specimens with higher exotic floral abundance (Fig. 4). It has been demonstrated that larger bee species are able to cover longer distances in the search of resources than smaller and medium sized bees (Greenleaf et al., 2007), but their success is still affected by the quality of their habitat, decreasing in sites were urbanization is stronger (Martins, Gonçalves & Melo, 2013). If exotic flora keeps expanding, it is possible there could be changes not only for bees, but also for this entire high-altitude ecosystem (Goulson & Nicholls, 2016). Different bee species may prefer different floral resources during foraging (Hinners & Hjelmroos-Koski, 2009; Harmon-Threatt & Kremen, 2015), and they can also have varied responses toward the use of exotic flora over native plant species (Morandin & Kremen, 2013). For an optimal larval development, bees need to reach their pollen nutritional requirements (Brodschneider & Crailsheim, 2010), therefore, low quality pollen can affect the development and survival of native bees and consequently affect the complete assemblage of this group of insects (Herbert, Bickley & Shimanuk, 1970; Peng & Jay, 1976; Roulston & Cane, 2002; Di Pasquale et al., 2013). Nevertheless, even if the abundance of small and medium native bees increased, the long-term effects of these changes on this native bee assemblage are still unknown and further studies are needed to assess their extent and implications.

The landscape variables didn’t show an effect over native bee richness and abundance. Considering our study was located in a small Andean urban area with a great number of “green spaces” (gardens and town squares) it could be possible that connectivity still remains unaffected. Regardless if urbanization results in a higher number of edifications coupled with destruction and fragmentation of natural habitats, loss of areas capable of sustaining wild life (McIntyre et al., 2001; Seto, Günerlap & Hutyra, 2012) and thus, habitat loss and permanent disappearance of wild species as a consequence (McKinney, 2002; Harrison, Gibbs & Winfree, 2018), these predictors will depend of the quality of the surrounding landscape (Tscharntke et al., 2005). Therefore, if “green spaces” are large or close from one another, the impact these areas would have in preserving biodiversity in the long-term may buffer the effects of urbanization (Rudd, Vala & Schaefer, 2002; Goddard, Dougill & Benton, 2010).

Given the response of the functional traits in comparison with native bee richness and abundance alterations due to changes in the landscape, the use of species-specific traits could be an important tool to detect early changes in native bee assemblages and take appropriate conservation measures. This work contributed to the scarce information regarding the connection between high-altitude pollinators and urbanization effects, especially in regards to the relationship between the introduction of exotic flora and native assemblages. Our work stresses the need to elucidate the direct effect exotic flora can have in native bee ecophysiology and the long-term ecological dynamics. This study also highlights the urgent need to plan urban expansion ahead of time, taking into account the biodiversity that will be affected so management measures can also be included. For instance, the control of weeds and introduction of exotic ornamental plant species. Furthermore, it is important to stress the need for science education and outreach to generate a common conscience of the value of local biodiversity and the ecosystem services they provide (Wilson, Forister & Carril, 2017). Biotic homogenization has been described as one of the most detrimental human activities on biological diversity (McKinney & Lockwood, 1999). This, in addition to the fact of the disconnection of our own species with nature (Navarro-Perez & Tidball, 2012), could make it more difficult for humans to empathize with biodiversity and promote its conservation (McKinney, 2006). A future goal should be to include management practices that buffer the effects of urbanization over biodiversity.

## Conclusions

Exotic flora in montane habitats could modify the composition of native bee assemblages. Nevertheless, further research is necessary in order to assess the ecological and evolutionary consequences of these invasions. Furthermore, we did not find a landscape effect from urbanization variables, we suggest that the occurrence of empty lots with remaining patches of native flora may contribute to habitat connectivity in this high-altitude town, reducing the effect of urbanization itself over native bee species. We propose that the existence of “green spaces,” composed by local plant community, and control of exotic plant species may ameliorate the effects of human expansion in high elevation bee habitats.

## Supplemental Information

10.7717/peerj.5916/supp-1 Supplemental Information 1 Native bee raw data. Raw data of native bee specimens collected during the two year study.

10.7717/peerj.5916/supp-2 Supplemental Information 2 Raw data for native bee diversity and landscape analysis. Raw data used for the analysis of the landscape variables over native bee richness and abundance.

10.7717/peerj.5916/supp-3 Supplemental Information 3 Raw data for assemblage analysis. Raw data used for the analysis of the relationship of exotic floral abundance over the high-altitude bee assemblage.

10.7717/peerj.5916/supp-4 Supplemental Information 4 Map of the distribution of the 8 selected sites for the study in Farellones, Chile. On the right side of the panel is a representation of Chilean territory highlighting our study site. On the upper right corner, a regional scale map also points where our study site was located. Main frame of this figure shows a satellite image of our study site. Symbols are explained on the lower right side of the figure. Map data: Gino Sandoval.

10.7717/peerj.5916/supp-5 Supplemental Information 5 List of native bee species collected during both seasons of the study, their functional traits and observed floral associations. Each column depicts information of each species collected during the study and the number of specimens collected for both pooled seasons.

10.7717/peerj.5916/supp-6 Supplemental Information 6 Spearman correlation coefficients between the landscape variables considered in the study. First column corresponds to each sampled season. The following columns and rows represent the spearman correlation value for the landscape variables considered for the study. Cells with significant p-value are written in bold.

10.7717/peerj.5916/supp-7 Supplemental Information 7 GLM results for mountain native bee richness and abundance depending on the landscape variables for each season of the study. Factor column represents the landscape variables of the study site and B represents the coefficient estimates.

10.7717/peerj.5916/supp-8 Supplemental Information 8 List of floral plant species registered during field work and their origin. Plant species collected at the study site during both seasons. The second and third columns represent their family and their origin for Chile.

## Additional Information and Declarations

### Data Availability

The following information was supplied regarding data availability: Villagra Gil CA. 2018. Along urbanization sprawl, exotic plants (Hymenoptera Apoidea) distort native bee assemblages in high elevation Andes ecosystem. Available at osf.io/qrphj.
