# Supplementary figures and images for: Along urbanization sprawl, exotic plants distort native bee (Hymenoptera: Apoidea) assemblages in high elevation Andes ecosystem
Source: PeerJ. 2018 Nov 7;6:e5916. doi: 10.7717/peerj.5916 (PMC6228549; doi:10.7717/peerj.5916)

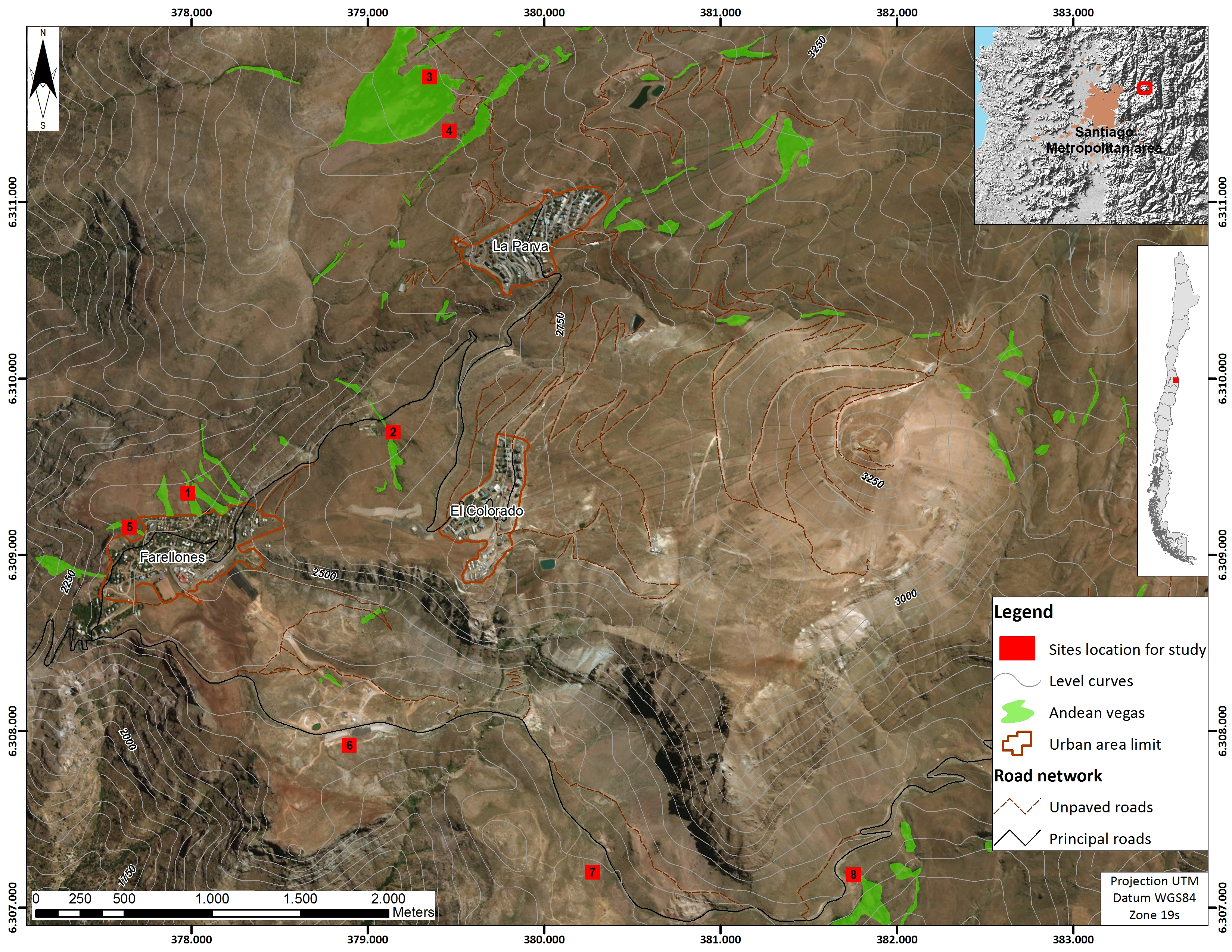

Supplement: Supplemental Information 4 — On the right side of the panel is a representation of Chilean territory highlighting our study site. On the upper right corner, a regional scale map also points where our study site was located. Main frame of this figure shows a satellite image of our study site. Symbols are explained on the lower right side of the figure. Map data: Gino Sandoval. [file peerj-06-5916-s004.png]
