# Supplementary material for: Along urbanization sprawl, exotic plants distort native bee (Hymenoptera: Apoidea) assemblages in high elevation Andes ecosystem
Source: PeerJ. 2018 Nov 7;6:e5916. doi: 10.7717/peerj.5916 (PMC6228549; doi:10.7717/peerj.5916)
Supplement: Supplemental Information 5 — Each column depicts information of each species collected during the study and the number of specimens collected for both pooled seasons. [file peerj-06-5916-s005.docx]

|  | | | | | | | |
| --- | --- | --- | --- | --- | --- | --- | --- |
| Specie | Family | Number of specimens | Body size | Feeding behavior^a^ | Parasitism | Observed floral associations during the study | References |
|  |  |  |  |  |  |  |  |
| *Acamptopoeum submetallicum* | Andrenidae | 25 | Medium | Polylectic | Non-parasitic | *Anthemis cotula* | Rozen 1967; Packer et al. 2005 |
|  |  |  |  |  |  |  |  |
| *Alloscirtetica gayi* | Apidae | 5 | Large | Polylectic | Non-parasitic | *A. cotula* | Wagenknecht 1970 |
|  |  |  |  |  |  | *Stachys philippiana* |  |
|  |  |  |  |  |  |  |  |
| *Alloscirtetica rufitarsis* | Apidae | 8 | Large | Polylectic | Non-parasitic | *Adesmia gracilis* | Wagenknecht 1970 |
|  |  |  |  |  |  | *A. cotula* |  |
|  |  |  |  |  |  | *S. philippiana* |  |
|  |  |  |  |  |  |  |  |
| *Anthidium chilense* | Megachilidae | 2 | Medium | Polylectic | Non-parasitic | NO | Toro & Rodríguez 1998 |
|  |  |  |  |  |  |  |  |
| *Anthidium chubuti* | Megachilidae | 16 | Large | Polylectic | Non-parasitic | *Adesmia* sp*.* | Toro & Rodríguez 1998 |
|  |  |  |  |  |  | *Chaetanthera pusilla* |  |
|  |  |  |  |  |  |  |  |
| *Anthidium espinosai* | Megachilidae | 1 | Large | *Polylectic* | Non-parasitic | NO | Toro & Rodríguez 1998 |
| *Anthophora incerta* | Apidae | 2 | Large | Polylectic | Non-parasitic | NO | Ruiz 1940 |
| *Bombus dahlbomii* | Apidae | 121 | Large | Polylectic | Non-parasitic | *A. cotula* | Abrahamovich et al. 2001 |
|  |  |  |  |  |  | *Chuquiraga oppositifolia* |  |
|  |  |  |  |  |  | *Phacelia secunda* |  |
|  |  |  |  |  |  | *S. philippiana* |  |
|  |  |  |  |  |  |  |  |
| *Cadeguala occidentalis* | Colletidae | 1 | Large | Polylectic | Non-parasitic | *S. philippiana* | Packer et al. 2005; Montalva et al. 2011 |
|  |  |  |  |  |  |  |  |
| *Caenohalictus aplacodes* | Halictidae | 3 | Medium | *Polylectic* | Non-parasitic | *Berberis empetrifolia* | Michener et al. 1979; Rojas & Toro 2000 |
| *Caenohalictus iodurus* | Halictidae | 117 | Small | Polylectic | Non-parasitic | *A. cotula* | Michener et al. 1979; Rojas & Toro 2000 |
|  |  |  |  |  |  | *B. empetrifolia* |  |
|  |  |  |  |  |  | *Taraxacum officinale* |  |
|  |  |  |  |  |  |  |  |
| *Caenohalictus rostraticeps* | Halictidae | 6 | Medium | Polylectic | Non-parasitic | *Alstromeria pallida* | Michener et al. 1979; Rojas & Toro 2000 |
|  |  |  |  |  |  | *Madia sativa* |  |
|  |  |  |  |  |  | *S. philippiana* |  |
|  |  |  |  |  |  |  |  |
| *Callistochlora chloris* | Halictidae | 61 | Medium | Polylectic | Non-parasitic | *A. cotula* | González-Vaquero & Galvani 2016 |
|  |  |  |  |  |  | *Brassica campestris* |  |
|  |  |  |  |  |  | *C. oppositifolia* |  |
|  |  |  |  |  |  | *T. officinale* |  |
|  |  |  |  |  |  |  |  |
| *Callistochlora prothysteres* | Halictidae | 1 | Medium | *Polylectic* | Non-parasitic | *A. cotula* | González-Vaquero & Galvani 2016 |
|  |  |  |  |  |  |  |  |
| *Caupolicana bicolor* | Colletidae | 1 | Large | *Polylectic* | Non-parasitic | NO | Ruiz 1938 |
|  |  |  |  |  |  |  |  |
| *Centris cineraria* | Apidae | 14 | Large | Polylectic | Non-parasitic | *A. gracilis* | Wagenknecht, 1971; Chiappa et al. 2000 |
|  |  |  |  |  |  | *S. philippiana* |  |
| *Centris nigerrima* | Apidae | 22 | Large | Polylectic | Non-parasitic | *A. pallida* | Wagenknecht, 1971; Chiappa et al. 2000 |
|  |  |  |  |  |  | *Calceolaria arachnoidea* |  |
|  |  |  |  |  |  | *C. oppositifolia* |  |
|  |  |  |  |  |  | *S. philippiana* |  |
|  |  |  |  |  |  |  |  |
| *Chalepogenus caeruleus* | Apidae | 2 | Medium | Polylectic | Non-parasitic | NO | Roig-Alsina 1999 |
| *Chilicola (Heteroediscelis) curvapeligrosa* | Colletidae | 34 | Small | *Polylectic* | Non-parasitic | NO | Jaffuel & Pirión 1926; González & Giraldo 2009 |
|  |  |  |  |  |  |  |  |
| *Colletes araucariae* | Colletidae | 41 | Medium | Polylectic | Non-parasitic | *A. cotula* | Ruiz 1944; Toro 1999 |
|  |  |  |  |  |  | *C. oppositifolia* |  |
|  |  |  |  |  |  | *M. sativa* |  |
|  |  |  |  |  |  | *P. secunda* |  |
|  |  |  |  |  |  |  |  |
| *Colletes fulvipes* | Colletidae | 2 | Large | *Polylectic* | Non-parasitic | *Adesmia* sp. | Ruiz 1944; Toro 1999 |
|  |  |  |  |  |  |  |  |
| *Colletes musculus* | Colletidae | 18 | Medium | Polylectic | Non-parasitic | *A. cotula* | Ruiz 1944; Toro 1999 |
|  |  |  |  |  |  | *B. empetrifolia* |  |
|  |  |  |  |  |  |  |  |
| *Diadasia chilensis* | Apidae | 22 | Medium | Polylectic | Non-parasitic | *A. cotula* | Ruiz 1940; Montalva et al. 2010 |
|  |  |  |  |  |  | *Senecio eruciformis* |  |
| *Epiclopus gayi* | Apidae | 1 | Large | Polylectic | Parasitic | NO | Wagenknecht 1969 |
| *Epiclopus lendlianus* | Apidae | 5 | Large | Polylectic | Parasitic | NO | Wagenknecht 1969 |
| *Euherbstia excellens* | Andrenidae | 1 | Large | *Polylectic* | Non-parasitic | *A. cotula* | Hurd & Linsley 1976; Rozen 1993 |
|  |  |  |  |  |  |  |  |
| *Isepeolus luctuosus* | Apidae | 1 | Medium | Polylectic | Parasitic | NO | Spinola 1851; Montalva et al. 2010 |
|  |  |  |  |  |  |  |  |
| *Kelita* sp. | Apidae | 4 | Small | NA | Parasitic | NO | Ehrenfeld & Rozen 1977 |
| *Lasioglossum* sp. | Halictidae | 279 | Small | Polylectic | Non-parasitic | *A. cotula* | Montalva et al. 2010; Polidori et al. 2010 |
|  |  |  |  |  |  | *S. philippiana* |  |
|  |  |  |  |  |  |  |  |
| *Liphanthus andinus* | Andrenidae | 50 | Small | *Oligolectic* | Non-parasitic | NO | Rozen 1967; Mena & Ruz 2003 |
| *Liphanthus coquimbensis* | Andrenidae | 6 | Small | *Oligolectic* | Non-parasitic | NO | Rozen 1967; Mena & Ruz 2003 |
| *Liphanthus sabulosus* | Andrenidae | 56 | Small | Oligolectic | Non-parasitic | NO | Rozen 1967; Mena & Ruz 2003 |
|  |  |  |  |  |  |  |  |
| *Megachile (Dasymegachile) distinguenda* | Megachilidae | 12 | Large | *Polylectic* | Non-parasitic | *Trifolium repens* | Durante et al. 2006 |
|  |  |  |  |  |  |  |  |
| *Megachile pollinosa* | Megachilidae | 1 | Large | Polylectic | Non-parasitic | NO | Raw 2007 |
|  |  |  |  |  |  |  |  |
| *Megachile saulcyi* | Megachilidae | 8 | Large | Polylectic | Non-parasitic | *A. pallida* | Durante et al. 2006 |
|  |  |  |  |  |  | *A. cotula* |  |
|  |  |  |  |  |  | *C. oppositifolia* |  |
|  |  |  |  |  |  |  |  |
| *Megachile semirufa* | Megachilidae | 35 | Large | Polylectic | Non-parasitic | *C. oppositifolia* | Durante et al. 2006; Montalva et al. 2012 |
|  |  |  |  |  |  | *P. secunda* |  |
|  |  |  |  |  |  | *Solidago chilensis* |  |
|  |  |  |  |  |  | *T. repens* |  |
|  |  |  |  |  |  |  |  |
| *Protandrena* sp. | Andrenidae | 7 | Small | *Polylectic* | Non-parasitic | NO | Gonzalez & Ruz, 2007; Gonzalez et al. 2013 |
|  |  |  |  |  |  |  |  |
| *Rhophitulus evansi* | Andrenidae | 30 | Small | Oligolectic | Non-parasitic | *S. eruciformis* | Ruz & Chiappa, 2004; Rozen 2014 |
|  |  |  |  |  |  | *S. chilensis* |  |
|  |  |  |  |  |  |  |  |
| *Ruizantheda cerdai* | Halictidae | 1 | Medium | *Polylectic* | Non-parasitic | NO | Spinola 1851; Jaffuel & Pirión 1926; Montalva et al. 2010 |
|  |  |  |  |  |  |  |  |
| *Ruizantheda mutabilis* | Halictidae | 5 | Medium | Polylectic | Non-parasitic | *A. cotula* | Spinola 1851; Jaffuel & Pirión 1926; Montalva et al. 2010 |
|  |  |  |  |  |  |  |  |
| *Ruizantheda nigrocaerulea* | Halictidae | 1 | Medium | Polylectic | Non-parasitic | *A. cotula* | Spinola 1851; Jaffuel & Pirión 1926; Montalva et al. 2010 |
|  |  |  |  |  |  |  |  |
| *Ruizantheda proxima* | Halictidae | 1 | Medium | *Polylectic* | Non-parasitic | *A. cotula* | Spinola 1851; Jaffuel & Pirión 1926; Montalva et al. 2010 |
|  |  |  |  |  |  |  |  |
| *Sphecodes rugulosus* | Halictidae | 3 | Small | *Polylectic* | Parasitic | *A. cotula* | Montalva et al. 2010; Özbek et al. 2015 |
| *Svastrides melanura* | Apidae | 4 | Large | Polylectic | Non-parasitic | *A. cotula* | Ruiz 1940; Wagenknecht 1970; Montalva et al. 2010 |
| *Trichothurgus herbsti* | Megachilidae | 1 | Large | Polylectic | Non-parasitic | NO | Walter & Sielfeld 1973 |
|  |  |  |  |  |  |  |  |
|  |  |  |  |  |  |  |  |
| *Xeromelissa* sp. | Colletidae | 15 | Small | *Polylectic* | Non-parasitic | NO | Rozen & Wyman 2015 |
| ^a^Data in italic represents when information available from the nearest related species was needed. | | | | | |  |  |
| NA: Not available |  |  |  |  |  |  |  |
| NO: Not observed |  |  |  |  |  |  |  |

Reference List:

Abrahamovich AH, Tellería MC, Díaz NB. 2001. Bombus species and their associated flora in Argentina. *Bee World* 82(2):76–87.

Chiappa E, Bascuñan R, Rodriguez S. 2000. Nidificación, Conducta De Machos De Centris (Wagenknechtia) Rodophthalma Pérez (Hymenoptera: Anthophoridae) Y Comparación Con Otras Especies Chilenas Del Género. *Acta Entomológica Chilena* 24:19–28.

Durante S, Abrahamovich A, Lucia M. 2006. El Subgénero Megachile (Dasymegachile) Mitchell Con Especial Referencia A Las Especies Argentinas (Hymenoptera: Megachilidae). *Neotropical Entomology* 35:791–802.

Gonzalez V, Giraldo C. 2009. New Andean bee species of Chilicola spinola (Hymenoptera: Colletidae, Xeromelissinae) with notes on their biology. *Caldasia* 31(1):145–154.

Gonzalez VH, Ruz L. 2007. New enigmatic Andean bee species of Protandrena (Hymenoptera, Andrenidae, Panurginae). Revista Brasileira de *Entomologia* 51(4):397–403 DOI 10.1590/s0085-56262007000400001.

Gonzalez VH, Engel MS, Sepúlveda PA. 2013. Taxonomic and biological notes on Andinopanurgus (Hymenoptera: Andrenidae). *Journal of Melittology* 3(1):1–10 DOI 10.17161/jom.v0i3.4437.

González-Vaquero RA, Galvani GL. 2016. Antennal sensilla analyses as useful tools in the revision of the sweat-bee subgenus Corynura (Callistochlora) Michener (Hymenoptera: Halictidae). *Zoologischer Anzeiger* 262:29–42 DOI 10.1016/j.jcz.2016.03.006.

Hurd PD, Linsley EG. 1976. The bee family Oxaeidae with a revision of the North American species (Hymenoptera: Apoidea). *Smithsonian Contributions to Zoology* 220:1–75 DOI 10.5479/si.00810282.220.

Mena P, Ruz L. 2003. Field observations on the behavior and nesting habits of Liphanthus sabulosus reed (Hymenoptera: Andrenidae). *Journal of the Kansas Entomological Society* 76(2):198–202.

Michener C, Breed M, Bell W. 1979. Seasonal cycles, nests, and social behavior of some Colombian halictine bees (Hyrmenoptera; Apoidea). *Revista de Biología Tropical* 27:13–34.

Montalva J, Castro B, Allendes J. 2012. Biología De Nidificación De Megachile semirufa (Hymenoptera: Megachilidae: Dasymegachile) En Alta Montaña, Chile. *Caldasia* 34:475–481.

Montalva J, Sepúlveda Y, Baeza R. 2011. Cadeguala occidentalis (Haliday, 1836) (Hymenoptera: Colletidae: Diphaglossinae): Biología De Nidificación Y Morfología De Los Estados Inmaduros*. Boletín De Biodiversidad De Chile* 5:3–21.

Özbek H, Bogusch P, Straka J. 2015. A contribution to the kleptoparasitic bees of Turkey: part I., the genus Sphecodes Latreille (Hymenoptera: Halictidae). *Turkish Journal of Zoology* 39:1095–1109 DOI 10.3906/zoo-1501-43.

Polidori C, Rubichi A, Barbieri V, Trombino L, Donegana M. 2010. Floral resources and nesting requirements of the ground-nesting social bee, Lasioglossum malachurum (Hymenoptera: Halictidae), in a Mediterranean semiagricultural landscape. *Psyche: A Journal of Entomology* 2010:1–11 DOI 10.1155/2010/851947.

Raw A. 2007. An annotated catalogue of the leafcutter and mason bees (genus Megachile) of the Neotropics. *Zootaxa* 1601:1–127.

Roig-Alsina A. 1999. Revisión De Las Abejas Colectoras De Aceites Del Género Chalepogenus Holmberg (Hymenoptera, Apidae, Tapinotaspidini). *Revista del Museo Argentino de Ciencias Naturales* 1:67–101.

Rojas F, Toro H. 2000. Revisión De Las Especies De Caenohalictus (Halictidae-Apoidea) Presentes En Chile. *Boletín del Museo Nacional de Historia Natural* 49:163–214.

Rozen JG. 2014. Nesting biology and immature stages of the panurgine bee genera rhophitulus and cephalurgus (Apoidea: Andrenidae: Protandrenini). *American Museum Novitates* 3814(3814):1–16 DOI 10.1206/3814.1.

Rozen JG, Wyman ES. 2015. The chilean bees xeromelissa nortina and X. sielfeldi: their nesting biologies and immature stages, including biological notes onX. rozeni (Colletidae: Xeromelissinae). *American Museum Novitates* 3838(3838):1–20 DOI 10.1206/3838.1.

Ruiz F. 1938. El género Caupolicana Spinola (Apidae, Hymen.). *Revista Chilena de Historia Natural* 42:39–55.

Ruiz F. 1940. Apidologia Chilena I Parte*. Revista Chilena de Historia Natural* 44:282–377.

Ruz L, Chiappa E. 2004. Protandrena evansi, a new panurgine bee from chile (Hymenoptera: Andrenidae). *Journal of The Kansas Entomological Society* 77(4):788–795 DOI 10.2317/e-41.1.

Spinola M. 1851. Himenópteros. In: Gay C, ed. Historia Física y Política de Chile. Vol. 6. Paris: Zoologia, 153–569.

Wagenknecht R. 1971. Contribución A La Biología De Los Apoidea Chilenos. *Anales Del Museo De Historia Natural* 4:277–286.

Walter H, Sielfeld K. 1973. Contribución al conocimiento de las especies chilenas del género Trichothurgus Moure 1949 (Himenoptera, apoidea). *Noticiario Mensual del Museo Nacional de Historia Natural* 206–207:3–10.
