# Supplementary material for: Along urbanization sprawl, exotic plants distort native bee (Hymenoptera: Apoidea) assemblages in high elevation Andes ecosystem
Source: PeerJ. 2018 Nov 7;6:e5916. doi: 10.7717/peerj.5916 (PMC6228549; doi:10.7717/peerj.5916)
Supplement: Supplemental Information 6 — First column corresponds to each sampled season. The following columns and rows represent the spearman correlation value for the landscape variables considered for the study. Cells with significant p-value are written in bold. [file peerj-06-5916-s006.docx]

|  | | | | | |
| --- | --- | --- | --- | --- | --- |
| 2016/2017 Season | Distance to nearest town (p-value) | Distance to roads (p-value) | Infraestructures (p-value) | Altitude (p-value) | Native floral abundance (p-value) |
| Distance to roads (p-value) | -0.262 (0.531) | - | - | - | - |
| Urban landscape (p-value) | 0.412 (0.310) | -0.247 (0.555) | - | - | - |
| Altitude (p-value) | -0.333 (0.420) | 0.262 (0.531) | -0.577 (0.134) | - | - |
| Native floral abundance (p-value) | -0.095 (0.823) | 0.310 (0.456) | 0.412 (0.310) | 0.333 (0.420) | - |
| Exotic floral abundance (p-value) | **-0.857 (0.007)** | 0.095 (0.823) | -0.247 (0.555) | 0.167 (0.693) | 0.119 (0.779) |
| 2017/2018 Season | Distance to nearest town (p-value) | Distance to roads (p-value) | Infraestructures (p-value) | Altitude (p-value) | Native floral abundance (p-value) |
| Distance to roads (p-value) | -0.262 (0.531) | - | - | - | - |
| Urban landscape (p-value) | 0.412 (0.310) | -0.247 (0.555) | - | - | - |
| Altitude (p-value) | -0.333 (0.420) | 0.262 (0.531) | -0.577 (0.134) | - | - |
| Native floral abundance (p-value) | 0.071 (0.867) | 0.167 (0.693) | 0.247 (0.555) | 0.476 (0.233) | - |
| Exotic floral abundance (p-value) | **-0.905 (0.002)** | 0.310 (0.456) | -0.412 (0.310) | 0.262 (0.531) | -0.143 (0.736) |
|  | |  |  |  |  |
