# Supplementary material for: Along urbanization sprawl, exotic plants distort native bee (Hymenoptera: Apoidea) assemblages in high elevation Andes ecosystem
Source: PeerJ. 2018 Nov 7;6:e5916. doi: 10.7717/peerj.5916 (PMC6228549; doi:10.7717/peerj.5916)
Supplement: Supplemental Information 7 — Factor column represents the landscape variables of the study site and B represents the coefficient estimates. [file peerj-06-5916-s007.docx]

|  | | | | |
| --- | --- | --- | --- | --- |
| Factor | B | Standard error | Wald Chi-Square | p-value |
| GLM: Richness season 2016/2017, Negative binomial distribution, AIC = 130.91 | | | | |
| Distance to nearest town | -0.001 | 0.0009 | 0.845 | 0.358 |
| Distance to roads | -0.001 | 0.0089 | 0.026 | 0.873 |
| Urban landscape | 0.003 | 0.0030 | 1.081 | 0.299 |
| Altitude | 0.000 | 0.0032 | 0.022 | 0.881 |
| Native floral abundance | 0.150 | 0.3334 | 0.202 | 0.653 |
| Factor | B | Standard error | Wald Chi-Square | p-value |
| GLM: Abundance season 2016/2017, Negative binomial distribution, AIC = 176.97 | | | | |
| Distance to nearest town | -0.001 | 0.0009 | 1.591 | 0.207 |
| Distance to roads | -0.002 | 0.0092 | 0.059 | 0.809 |
| Urban landscape | 0.002 | 0.0029 | 0.587 | 0.444 |
| Altitude | -0.001 | 0.0031 | 0.106 | 0.744 |
| Native floral abundance | 0.049 | 0.3044 | 0.026 | 0.871 |
| Factor | B | Standard error | Wald Chi-Square | p-value |
| GLM: Richness season 2017/2018, Negative binomial distribution, AIC = 130.91 | | | | |
| Distance to nearest town | -0.001 | 0.0008 | 0.604 | 0.437 |
| Distance to roads | -0.002 | 0.0081 | 0.062 | 0.803 |
| Urban landscape | 0.001 | 0.0027 | 0.284 | 0.594 |
| Altitude | 0.000 | 0.0033 | 0.005 | 0.943 |
| Native floral abundance | -0.067 | 0.3080 | 0.047 | 0.828 |
| Factor | B | Standard error | Wald Chi-Square | p-value |
| GLM: Abundance season 2017/2018, Negative binomial distribution, AIC = 176.97 | | | | |
| Distance to nearest town | -0.001 | 0.0008 | 1.597 | 0.206 |
| Distance to roads | 0.003 | 0.0080 | 0.175 | 0.676 |
| Urban landscape | 0.002 | 0.0026 | 0.448 | 0.503 |
| Altitude | -0.001 | 0.0033 | 0.048 | 0.827 |
| Native floral abundance | -0.123 | 0.2985 | 0.170 | 0.680 |
| Results of generalized linear models with abundance or species richness as dependent variables and landscape variables as independent variables. The effect of independent variables was nested in the year to account for interannual. | | | | |
| *AIC=Akaike Information Criterion. | | | | |
