# Supplementary material for: Along urbanization sprawl, exotic plants distort native bee (Hymenoptera: Apoidea) assemblages in high elevation Andes ecosystem
Source: PeerJ. 2018 Nov 7;6:e5916. doi: 10.7717/peerj.5916 (PMC6228549; doi:10.7717/peerj.5916)
Supplement: Supplemental Information 8 — Plant species collected at the study site during both seasons. The second and third columns represent their family and their origin for Chile. [file peerj-06-5916-s008.docx]

|  | | |
| --- | --- | --- |
| Species | Family | Origin |
| *Adesmia gracilis* | Fabaceae | Native |
| *Adesmia* sp. | Fabaceae | Native |
| *Alstroemeria pallida* | Alstroemeriaceae | Native |
| *Anthemis cotula* | Asteraceae | Exotic |
| *Astragalus looseri* | Fabaceae | Native |
| *Berberis empetrifolia* | Berberidaceae | Native |
| *Brassica campestris* | Brassicaceae | Exotic |
| *Calceolaria arachnoidea* | Calceolariaceae | Native |
| *Cardamine vulgaris* | Brassicaceae | Native |
| *Calandrinia affinis* | Montiaceae | Native |
| *Cerastium arvense* | Caryophyllaceae | Exotic |
| *Chaetanthera chilensis* | Asteraceae | Native |
| *Chaetanthera linearis* | Asteraceae | Native |
| *Chaetanthera pusilla* | Asteraceae | Native |
| *Chuquiraga oppositifolia* | Asteraceae | Native |
| *Convolvulus arvensis* | Convolvulaceae | Exotic |
| *Cynoglossum creticum* | Boraginaceae | Exotic |
| *Epilobium nivale* | Onagraceae | Native |
| *Gilia crassifolia* | Polemoniaceae | Native |
| *Haplopappus diplopappus* | Asteraceae | Native |
| *Haplopappus schumannii* | Asteraceae | Native |
| *Lobelia oligophylla* | Campanulaceae | Native |
| *Madia sativa* | Asteraceae | Native |
| *Microsteris gracilis* | Polemoniaceae | Native |
| *Mutisia sinuata* | Asteraceae | Native |
| *Oenothera acaulis* | Onagraceae | Endemic |
| *Olsynium philippii* | Iridaceae | Native |
| *Perezia carthamoides* | Asteraceae | Native |
| *Phacelia secunda* | Boraginaceae | Native |
| *Quinchamalium chilense* | Santalaceae | Native |
| *Rhodophiala rhodolirion* | Amaryllidaceae | Native |
| *Sanicula graveolens* | Apiaceae | Native |
| *Schizanthus hookeri* | Solanaceae | Native |
| *Senecio eruciformis* | Asteraceae | Native |
| *Solidago chilensis* | Asteraceae | Exotic |
| *Stachys philippiana* | Lamiaceae | Endemic |
| *Taraxacum officinale* | Asteraceae | Exotic |
| *Trifolium repens* | Fabaceae | Exotic |
| *Veronica anagallis-aquatica* | Plantaginaceae | Exotic |
